# A New Dual-Frequency Liquid Crystal Lens with Ring-and-Pie Electrodes and a Driving Scheme to Prevent Disclination Lines and Improve Recovery Time

**DOI:** 10.3390/s110505402

**Published:** 2011-05-18

**Authors:** Yung-Yuan Kao, Paul C.-P. Chao

**Affiliations:** 1 Department of Electrical Engineering, National Chaio Tung University, Hsinchu, 30010, Taiwan; E-Mail: yungyuan.kao@gmail.com; 2 Institute of Imaging and Biophotonics, National Chaio Tung University, Tainan, 30010, Taiwan

**Keywords:** Dual-Frequency Liquid Crystals (DFLC), tunable LC lens, disclination lines, ring and pie electrodes

## Abstract

A new liquid crystal lens design is proposed to improve the recovery time with a ring-and-pie electrode pattern through a suitable driving scheme and using dual-frequency liquid crystals (DFLC) MLC-2048. Compared with the conventional single hole-type liquid crystal lens, this new structure of the DFLC lens is composed of only two ITO glasses, one of which is designed with the ring-and-pie pattern. For this device, one can control the orientation of liquid crystal directors via a three-stage switching procedure on the particularly-designed ring-and-pie electrode pattern. This aims to eliminate the disclination lines, and using different drive frequencies to reduce the recovery time to be less than 5 seconds. The proposed DFLC lens is shown effective in reducing recovery time, and then serves well as a potential device in places of the conventional lenses with fixed focus lengths and the conventional LC lens with a single circular-hole electrode pattern.

## 1. Introduction

Optical lenses have been used in many commercial imaging modules or communication devices such as mobile phones to realize the functions of focusing and/or zooming. In recent years, the capability of on-line tunable focal length in a conventional variable focus lens module has commonly been achieved by mechanical motion methods in compact camera modules (CCMs). The lens is actuated by stepping motors, piezo or voice coil motors (VCMs). Nevertheless, the control mechanism for lens motion is not only very complicated but also requires substantial space for lens movement. To remedy these problems, several past studies on the liquid crystal (LC) lenses with tunable focal length are proposed [1–5] with the aim of using them in the auto-focusing CCMs of mobile phones.

The LC lens was first introduced by Sato in 1979 [1]. The lens is shaped like a plano-convex or concave lens with the focal length varying between *f*_e_ and *f*_o_, which corresponds to the effective focal lengths for the extraordinary and ordinary rays. A hole-patterned indium-tin oxide (ITO-) electrode along with a counter-electrode were presented later on an LC lens by [6] to generate a non-uniform electrical field for focusing effects. Following [6], there were a number of works [6–18] presenting variations of LC lens designs with different electrode shapes, like circular, cylindrical, and hexagonal-hole-patterned ones. Some others adopted the design principle of the Fresnel zone plate [19,20], but rendering less focusing power from higher-order focused lights. Except for those in zone plates, all the studies are devoted for a better refractive index distribution (as close as possible to the GRIN lens [21–26]) and/or decreasing applied voltage level. Some works used high resistance materials to form the electrodes [27].

A new type of the dual-frequency LC (DFLC) lens with ring-and-pie electrodes and a suitable driving scheme is proposed in this study to reduce addressing time. The designed DFLC lens is aimed for applications in a compact camera module (CCM) with theoretically acceptable imaging quality. For cell phone applications, the suitable lens aperture is generally around 2 mm for a camera module with 2M∼5M pixels. The smallest focus length required should be less than 10 cm. It is pertinent to note that for CCM applications, polymer network liquid crystal lens (PNLC) lenses [28] and liquid lenses [29] are potential competitors to the LC lens. However, for PNLC lenses [28], they are unable to achieve the desired small focus length of less than 10 cm for compact camera modules (CCMs). As for liquid lenses [29], they are prone to high voltage, liquid leakage and difficulty to stabilize optical axis and image quality. However, they do generally have faster responses than LC lenses.

It is known that due to the thick LC layer [6–18] required to ensure enough focusing power in CCMs, the recovery time of the LC lens is generally very slow [30], and usually exceeds ten seconds. One remedy is to apply a high initial voltage. However, strong fields in the LC layer usually result in the occurrence of disclination lines. Ye *et al.* [31] illustrated a driving scheme to avoid disclination lines by applying an in-plane electric field in the LC layer, but it still has a long recovery time and required additional design work to determine drive voltages. In this study, aimed at preventing the disclination lines and improving the recovery time, a DFLC lens is proposed with ring and pie electrodes covering the entire aperture of the lens, in addition to the hole electrode in a conventional LC lens. Along with these new electrodes, a new driving scheme is forged with an in-plane electric field at some stage to prevent the occurrence of disclination. Furthermore, utilization of dual-frequency LC material MLC-2048 and driving at different frequencies enables a significant decrease in the recovery time, to less than 5 seconds. Note that the dual-frequency LC materials were also adopted in [32,33]. In [32], a deposited SiO_x_ layer is adopted to enforce high pre-tilt angles as 45 degree in order to decease recovery time. With large pre-tilt angles of the LC molecules at boundaries of the lens, the angular difference between the molecules at the center and boundaries of the LC column is small, leading to smaller effective index anisotropy, Δ*n*_eff_, along each LC column across the lens thickness. Based on LC lens theory, this results in a large, unsatisfactory minimum focus length for the aperture size of 2 mm in CCMs. Besides, this is not a reliable approach since it is difficult to precisely control pre-tilt angles. On the other hand, in [33] the lens was designed for a 3D volumetric display with a much larger focus length range than those required for CCMs.

This paper is organized in the following sections. Section 2 states the proposed design of a DFLC lens in the aspects of structure, ability to eliminating disclination and driving scheme. Section 3 provides details on fabrication while Section 4 describes experimental system, conduction and present resulted data. Section 5 concludes this work.

## 2. Design Principles

### 2.1. Lens Design

A conventional hole-type LC lens is designed to be equipped with a hole-ITO-electrode at the top of the upper glasses [1]. This structure forms an electric field in a normal distribution which has the weakest electrical intensity at the central region of the electrode’s hole. The liquid crystal molecules are orientated by the electric field cross the LC cell, and also aligned on the rubbed surfaces, *i.e.*, the inner surfaces of upper and lower glasses, with their axes parallel to the rubbing directions. In this study, the rubbing directions of two surfaces are designed identical. This design is widely known as antiparallel alignment. Due to the optical property, the birefringence index of liquid crystal, the distribution of LC molecules could lead to a gradient index under the electric field. This gradient index (GRIN) LC lens thus determines the focusing capability, evidenced from the fact that the optical path length (OPL) through the LC layer is the shortest along central optical axis and the largest on the edge axis of the lens.

**Figure 1.**
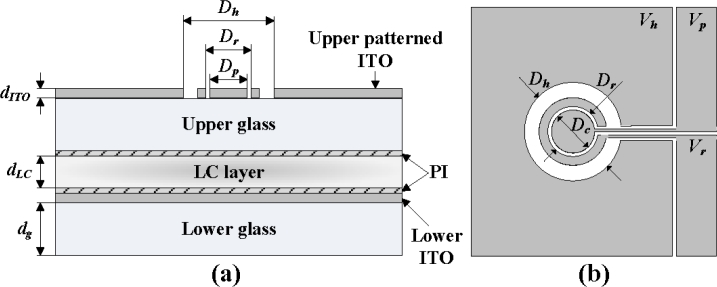
**(a)** Side-view of the ring-and-pie LC lens structure. **(b)** Top view.

The proposed design, as shown in Figure 1, has a pie electrode inside of a ring electrode, which is 2 mm in diameter (*D_r_*). The ring and pie electrodes offer a uniform distribution electric field in the LC layer as the designed driving scheme applies, specifically in the rising and recovering stages.

### 2.2. Eliminating Disclination Lines

**Figure 2.**
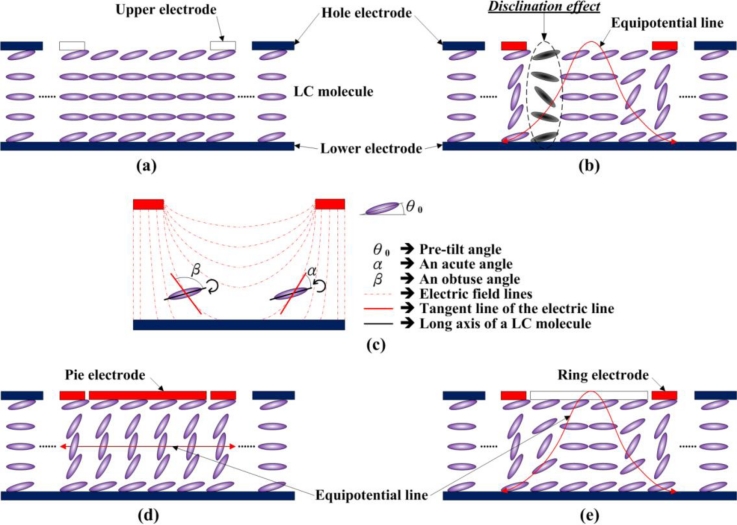
**(a)** The illustration of LC molecules in the conventional LC lens without voltage applied. **(b)** Disclination lines occurrence. **(c)** Electric fields and rotated directions of LC molecules. **(d)** In-plane electric field applied. **(e)** Disclination lines prevented.

Figure 2(a) illustrates the LC director distribution of a hole-type conventional LC lens, where the LCs are assumed positive type for illustration. It is seen from this figure that the LC molecules, anchored at the boundaries, are pre-tilted with anti-clockwise directors. With no driving voltage applied, the LC molecules are originally aligned as shown in Figure 2(a). However, disclination lines may occur in the conventional LC lens with a driving voltage applied, as shown in Figure 2(b). The reasons are given in the following: with an electric field in a typical hole-type LC lens generated, as shown in the Figure 2(c), the positive-type LC molecules tend to rotate to finally align in parallel with the field direction. While rotating, LC molecules tend to close the smaller angle (acute angle) between the long axis of LC molecules and the tangent line of the electric field lines. For most LC molecules, the rotations to close the acute angle are counterclockwise. However, for a few LC molecules as shown on the left hand side of Figure 2(c), the rotations are clockwise, finally resulting in final tilting states that are discontinuous to neighboring LCs, as shown in Figure 2(b).

Therefore, a driving algorithm is developed in this study for the ring-and-pie electrode to prevent the occurrence of disclination lines. This algorithm generally first applies a uniform in-plane electrode field across the LC lens by applying the same voltages to its ring and pie electrodes, as shown in Figure 2(d). In the second stage, as shown in Figure 2(e), the voltage on the pie electrode is floated to form a gradient electrical field, in order to render non-uniform rotations of LC molecules for focusing effect. In this way, the DFLC molecules are rotated to identical directions in the final stage in an anti-clockwise way. The disclination lines are then prevented by the ring-and-pie LC lens with this particular driving scheme.

### 2.3. Driving Scheme

**Figure 3.**
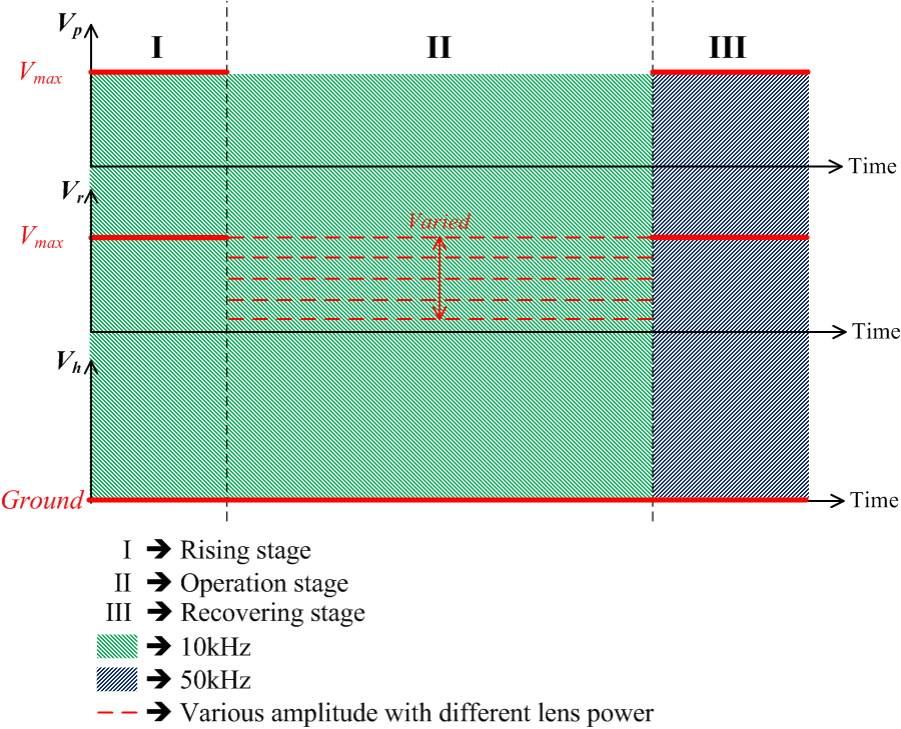
The driving scheme for the proposed DFLC lens.

The details of the proposed driving scheme for the proposed lens are stated in this subsection, which is, as shown in Figure 3, different from the conventional hole-type LC lens. With the designed two electrodes, ring and pie electrodes, for the DFLC lens, the application of driving and control signals for the DFLC lens structure can be divided into three stages which aim at preventing the disclination lines, focusing the lens and improving the recovery time, respectively. Note that the hole electrode is grounded all the time in order to generate the desired electric field across the lens thickness, especially in the fringe area of the lens.

In the rising stage, ring voltage is increased to the maximum driving AC voltage in 1 kHz, *Vmax*, as shown in Figure 3. In the meantime, the pie voltage is set equal to this maximum ring voltage for preventing disclination. An equal and uniform electric field is then generated across the LC cell layer to orientate all the DFLC molecules to certain angles. Thus it can prevent the occurrence of the disclination lines due to non-zero pre-tilt LC angles from the rubbing process. In the operating stage, the pie electrode is floated while the ring voltage varies for different lens powers. For fast switching from an arbitrary focal state to another arbitrary focal state, the basic drive scheme proposed in the present study, as shown in Figure 3, can also be adopted with slight modifications. For increasing focusing power, the drive voltage in stage II can be increased directly without operating through stages I and III to reach the desired focusing state in a short time. As for deceasing focusing power when LC recovery is involved (a much longer process than the increase of focusing power), the drive voltage in stage II is decreased in a complete addressing process to shorten the addressing time period. Finally, in the recovery stage, the pie electrode is re-applied with maximum driving AC voltage in 50 kHz to increase LC recovery speed. In this stage, the pie voltage is then equal to the ring maximum voltage in 50 kHz. It is pertinent to note that the high drive frequency as 50 kHz would possibly cause dielectric heating effects. These effects have been considered both theoretically and experimentally in [34–36]. However, in this study, the voltage in 50 kHz is applied only for the recovery stage, the time span of which is normally short as compared to the whole addressing operations, leading to no heating effect.

## 3. Fabrication

**Figure 4.**
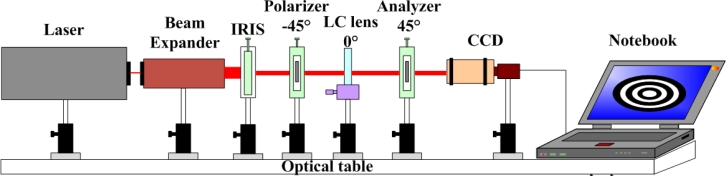
The experimental setup.

The structure and fabrication process of the ring and pie electrodes on a glass substrate for the proposed DFLC lens are described as follows: The experimental parameters and the structure specifications of the proposed lens are listed in Table 1. Two 0.55 mm glasses (*d_g_*) with 94% transmittance, are coated with 450 Å ITO (*d_ITO_*) and then stacked with a 50 μm Mylar spacer (*d_LC_*) in between. One of ITO glasses is etched with the desired ring-and-pie pattern. The polyimide (PI), AL-1426B, from Daily-polymer Corp. is next coated on these two glasses. Both PI layers are rubbed to form micro-grooved surfaces and render LC pre-tilts of 2°. The two glasses are then stacked with 50 μm Mylar spacer in between. The particular LC material MLC-2048 (a dual-frequency LC, DFLC) from Merck, the parameters of which are listed in Table 2, is filled into the space between two glasses. This DFLC has a cross-over frequency of 10 kHz, birefringence of Δn, 0.221, dielectric anisotropy of Δɛ = 3.3 at frequency 1 kHz and Δɛ = −3.0 at 50 kHz at room temperature. The gap between pie and ring electrodes is designed as 15 μm, and the ring width is 50 μm. This 15 μm corresponds to the minimum fabrication resolution of the process adopted. Figure 4 shows a photo of the fabricated ring-and-pie LC lens with four electrode pads—one for the lower ITO electrode which is always grounded during operation, one for the hole electrode, one for the upper ring electrode and the remaining one for the pie electrode. Note that the two ITO patterned glasses are stacked and the overall thickness of the proposed ring-and-pie type DFLC lens is only about 1.2 mm, which is extremely thin for being equipped to an auto-focusing module in a commercial mobile camera device.

## 4. Experimental

### 4.1. Interference Patterns

**Figure 5.**
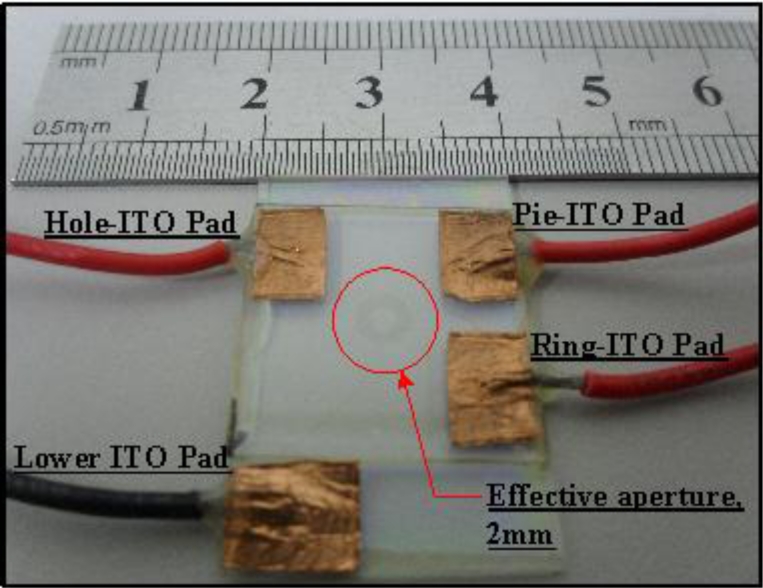
The photo of the proposed ring-and-pie DFLC lens.

With the designed GRIN DFLC lens fabricated, experiments were conducted next to validate the expected performance. Figure 4 illustrates the experimental setup for observing the focusing quality of the fabricated DFLC lens, as shown in Figure 5. A He-Ne laser of wavelength 632.8 nm is utilized as the light source. The emitted light beam passes through a 20X beam expander, an iris, a polarizer, the GRIN DFLC lens in radius 2 mm, an analyzer and finally reaches a charge-coupled device (CCD). The iris is used for trimming the incident light from the laser to observe the interference pattern easily. The polarizing direction of the polarizer, rubbing direction of the PI layer and that of the analyzer are in 45° difference subsequently, in order to generate desired circular interference patterns. Finally, for a clear interference pattern in the CCD, the DFLC lens is placed at the focal point of the lens module of the CCD.

**Figure 6.**
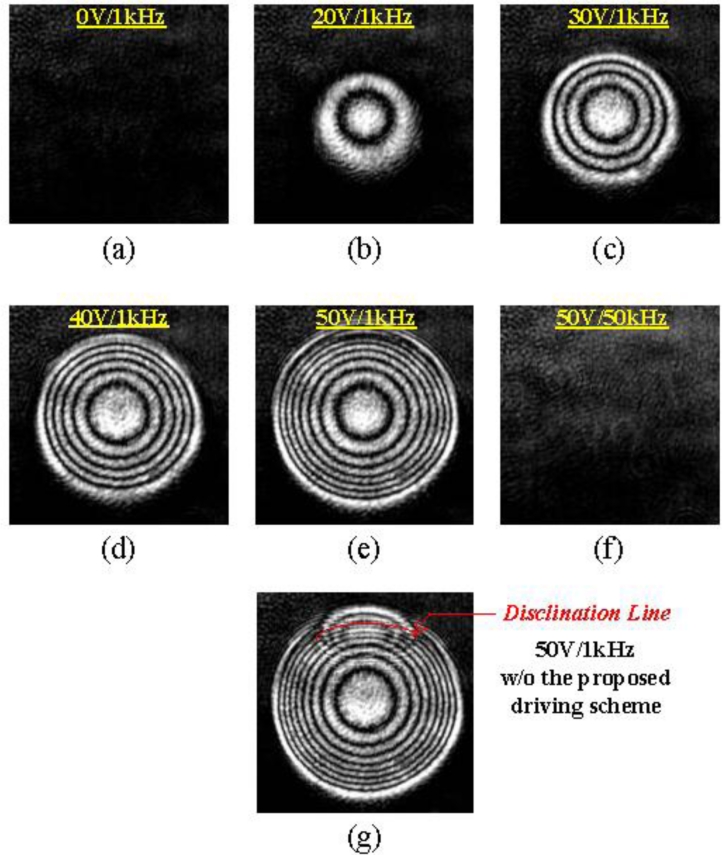
Interference patterns with the lens aperture in 2 mm **(a**–**e)** with the applied voltage varying from 0 V to 50 V. **(f)** with 50 V/50 k Hz applied voltage. **(g)** with disclination line.

Figure 6(a–e) shows the experimental interference patterns of the proposed DFLC lens with different applied voltages: 0 V, 20 V, 30 V, 40 V and 50 V at 1 kHz, respectively. It is clearly seen from Figure 6(a) to 6(e) that the interference rings are more densely present as the applied voltage is increasing, rendering shorter focus lengths of the DFLC lens. On the other hand, Figure 6(f) shows no interference rings, thus no focusing, since with 50 V in 50 kHz driving the dual-frequency LCs act like negative-type LC molecules. Finally, Figure 6(g) presents the interference with pie electrode floated, thus disclination line occurs.

**Figure 7.**
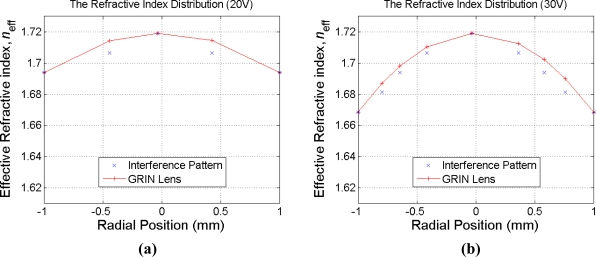

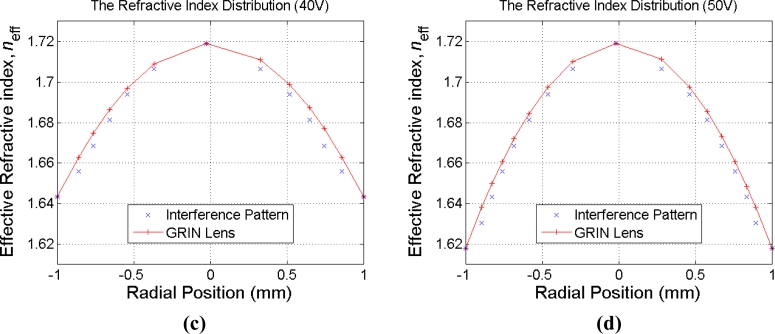
Extracted indices and focus lengths with 20 V, 30 V, 40 V and 50 V applied to the ring electrodes.

The interference patterns shown in Figure 6(b,c) are next deciphered to extract the focus lengths with different applied voltages. Note first that the set of polarizer-LC lens-analyzer in the experiment shown in Figure 5 presents a standard crossed-polarizer setup for the LC lens. Based on basic liquid crystal optics [37], the phase delay between two adjacent bright or dark rings in Figure 7 corresponds to a phase change of 2π. Thus, the resulted effective index difference between these two rings can be calculated by:

(1) $$\Delta n_{\text{eff}}=\frac{\lambda }{d}.$$

where Δ*n*_eff_ is defined as the effective anisotropy across LC lens thickness along an LC column [38], which is:

(2) $$\Delta n_{\text{eff}}(r)d=R,$$

where *r* is the radial position of the lens, *d* is the lens thickness and:

(3) $$R=\int_{z=0}^{d}\Delta n(z)dz$$

is the total optical retardation through an LC column. Also in Equation (3), Δ*n* is the optical anisotropy of a single LC molecule.

**Figure 8.**
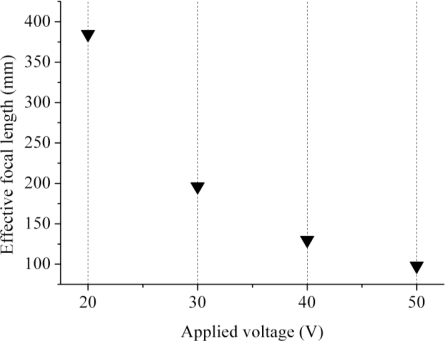
The focal length of the purposed LC lens with applied voltages, 20 V, 30 V, 40 V and 50 V.

With the above index difference between adjacent bright rings in hands and the fact that the effective index of the central solid circle in Figure 7 corresponds to *n*_eff_ = *n*_e_ = 1.7192, the indices at varied radial positions of the LC lens can be derived, as shown in Figure 7. Assuming the LC lens is acting like an gradient index lens (GRIN), the derived indices are fitted to a parabolic curve for estimating the focus length of the lens. The results are shown in Figure 8. It is seen that the dynamic range of the focus length 98 to 375 mm. The focus lengths herein based on the fitted data are regarded as and actually should be very close to actual focal lengths.

### 4.2. Response Time

**Figure 9.**
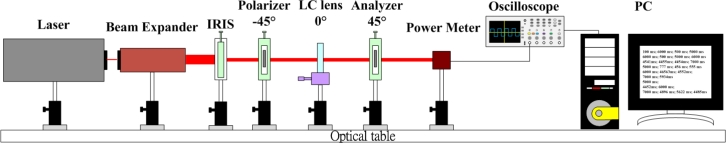
The experiment system with a power meter.

**Figure 10.**
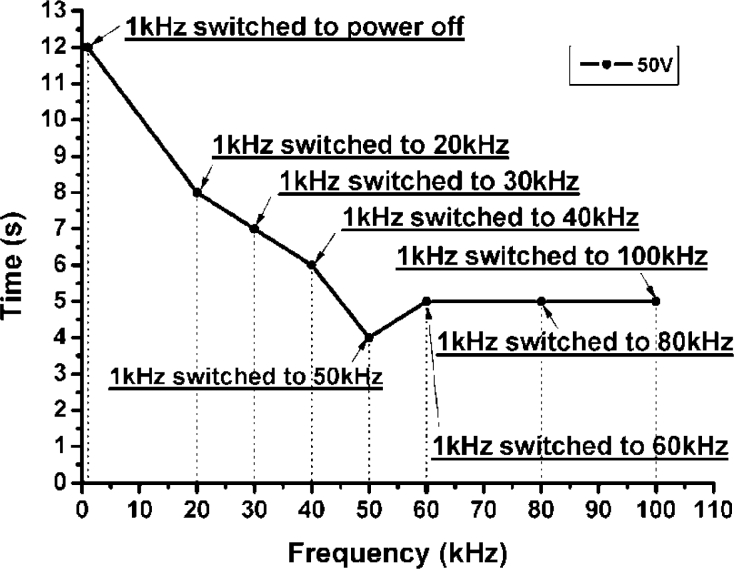
The recovery time of the ring-and-pie type DFLC lens at different driving frequencies.

**Figure 11.**
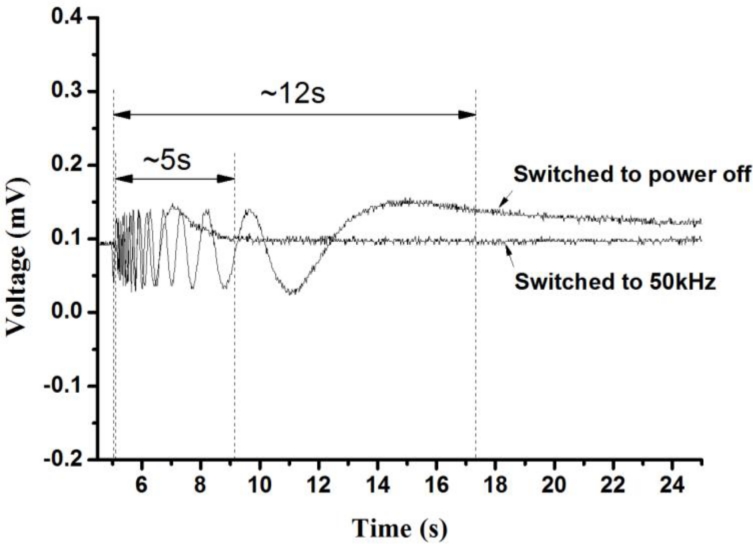
Output signal of the power meter in mV illustrating recovery times for the two cases with power-off and driving frequency in 50 kHz.

A similar experiment system with a power meter instead of a CCD is built to characterize response times in different stages, as shown in Figure 9. The data are relayed to a PC for analysis. The driving voltage is fixed to 50 V with the resulted EFL equal to 76.9 mm. As shown in Figure 10, where “1 kHz switched to power off” means that the applied voltage is changed from 1 kHz to power off, while the others are changed from 1 kHz to varied frequencies. The minimum recovery time occurs while the frequency is changed from 1 kHz to 50 kHz. This is because at 50 kHz the dielectric constant, −3.3, (see Table 2) of DFLC becomes negative and reaches its maximum magnitude. Thus, the molecule-turning dynamics of DFLC reaches the fastest state, leading to a minimum recovery time. On the other hand, Figure 11 shows the optical transmission of the DFLC lens measured by a power meter for the two different cases. It is found that the recovery time is significantly reduced to less than 5 seconds when the driving frequency is changed from 1 kHz to 50 kHz. As for the baseline case (without the proposed driving scheme applied but also with 1 kHz switched to power off), the recovery time is up to 12 seconds. Note that the conventional hole-type LC lens with double thickness in 100 μm (although not 50 μm in this study) as presented in [30] requires more than 50 seconds for the recovery time. It is shown that the proposed DFLC lens along with the new driving scheme is capable of shortening the recovery time significantly.

## 5. Conclusions

A new driving scheme design for the LC lens is proposed to prevent disclination lines and to improve the response time of LC lenses, particularly the recovery time. The designed DFLC lens has electrodes in a ring-and-pie electrode pattern and uses the MLC-2048 dual-frequency LC material to achieve the focusing function. The proposed DFLC lens is able to generate electrical fields leading to desired measured interference patterns, thus giving the same focusing capability as conventional LC lenses. Finally, the proposed DFLC lenses were fabricated and show superiority in terms of lens structure, electrodes and shortened response time with the new driving scheme. It is evidenced from experimental results that the recovery time of the proposed DFLC lens is successfully reduced to less than 5 seconds, a significant reduction compared to past studies.

## Figures and Tables

**Table 1.** The parameters and structure specifications of the proposed DFLC lens.

**Dimensions of the LC lens structure**		

**Symbol**	**Parameters**	**Value/Unit**

*d_ITO_*	The thickness of ITO	45 nm
*d_g_*	The thickness of glass	0.55 mm
*d_LC_*	The thickness of Mylar spacer (Cell gap)	50 μm
*D_h_*	The aperture of hole electrode	4 mm
*D_r_*	The aperture of ring electrode	2 mm
*D_p_*	The aperture of pie electrode	1.97 mm
-	The width of the ring electrode	50 μm
-	The distance between ring and pie electrodes	15 μm
-	The pre-tile angle	2 deg

**Table 2.** The parameters for DFLC, MLC-2048.

**Parameters of LC, MLC-2048, at 20 °C**		

**Symbol**	**Parameters**	**Value/Unit**

*n_e_*	The refractive index of e-ray	1.7192
*n_o_*	The refractive index of o-ray	1.4978
Δn	Optical anisotropy	0.2214
ɛ_//_	Dielectric constants of parallel to the director, at 1 kHz	10.6 F/m
ɛ_⊥_	Dielectric constants of perpendicular to the director, at 1 kHz	7.3 F/m
Δɛ	Dielectric at frequency 1 kHz	3.3
Δɛ	Dielectric at frequency 50 kHz	−3.3
